# Development of Immune-Specific Interaction Potentials and Their Application in the Multi-Agent-System VaccImm

**DOI:** 10.1371/journal.pone.0023257

**Published:** 2011-08-17

**Authors:** Anna Lena Woelke, Joachim von Eichborn, Manuela S. Murgueitio, Catherine L. Worth, Filippo Castiglione, Robert Preissner

**Affiliations:** 1 Institute for Physiology, Charité Universitätsmedizin Berlin, Berlin, Germany; 2 Institute for Computing Applications, National Research Council of Italy, Rome, Italy; Virginia Tech, United States of America

## Abstract

Peptide vaccination in cancer therapy is a promising alternative to conventional methods. However, the parameters for this personalized treatment are difficult to access experimentally. In this respect, *in silico* models can help to narrow down the parameter space or to explain certain phenomena at a systems level. Herein, we develop two empirical interaction potentials specific to B-cell and T-cell receptor complexes and validate their applicability in comparison to a more general potential. The interaction potentials are applied to the model VaccImm which simulates the immune response against solid tumors under peptide vaccination therapy. This multi-agent system is derived from another immune system simulator (C-ImmSim) and now includes a module that enables the amino acid sequence of immune receptors and their ligands to be taken into account. The multi-agent approach is combined with approved methods for prediction of major histocompatibility complex (MHC)-binding peptides and the newly developed interaction potentials. In the analysis, we critically assess the impact of the different modules on the simulation with VaccImm and how they influence each other. In addition, we explore the reasons for failures in inducing an immune response by examining the activation states of the immune cell populations in detail.

In summary, the present work introduces immune-specific interaction potentials and their application to the agent-based model VaccImm which simulates peptide vaccination in cancer therapy.

## Introduction

Cancer is still one of the major causes of death in industrial nations, although in principle the immune system is able to eradicate a tumor. Bearing that in mind, many studies have tried to trigger an anticancer immune response using different methods, e.g. adoptive cell transfer, cytokine therapy or vaccination schedules [1]. Immune therapy is promising, but its success has been limited so far. The main reason is that the mechanisms of the tumor-immune-interplay are still poorly understood. A huge amount of, sometimes conflicting, data has accumulated, which can be difficult to interpret. Therefore, it is desirable to have a simplified model able to highlight at the system level the main processes of the phenomenon. In addition, *in silico* experiments are far less expensive, less time consuming and a lot more flexible in terms of parameter changes.

We have described the main theoretical modeling techniques, differential equations and rule-based models, and their application to tumor immunology elsewhere [2]. For this project, we have chosen a rule-based model because of its capability to characterize every single cell or molecule in its location, developmental state and specificity.

The aim of our present study is to support peptide vaccination approaches in cancer therapy by modeling the specific tumor-immune interaction in a realistic fashion. For that purpose, we integrated a previously published model of the tumor-immune interplay [3] with a detailed description of the immune receptor-ligand interactions based on structural and sequence information. To our knowledge, this is the first approach simulating peptide vaccination in cancer treatment that takes the peptide sequence into account explicitly. An analogical approach designed for generic infections has been described by Rapin et al. [4].

### Rule-Based Modeling for Simulating the Immune System

Rule-based models are composed of discrete agents identifiable within a spatial environment. The agents interact, move and change their state according to behavioral rules in discrete time steps.

One of the first approaches to simulating the immune system using a cellular automaton was introduced in 1992 by Celada and Seiden [5]. Their cellular automaton called ImmSim used very simple rules but was able to reproduce several phenomena in immunology, e.g. clonal expansion of B- and T-cells after stimulation or the different time-lines of the first and second immunization. To account for specificity of the immune receptors, they developed a representation in the form of bit-strings that had to be complementary to favor an interaction between the immune cells [6]. Within the model, they examined optimal ranges to induce a sufficient immune response for some generic parameters such as the number of major histocompatibility complexes (MHCs) per individual or the number of self-peptides compared to the whole diversity of protein sequences. The ImmSim model has been extended and improved by several research groups (see below); the present study itself is based on an implementation of the Celada-Seiden ImmSim automaton.

Since 1992 the agent-based modeling community in theoretical biology has grown. Apart from ImmSim, other rule-based immune simulations have recently been developed which are briefly reviewed here.

The Basic Immune Simulator aims at understanding the interplay between the innate and the adaptive immune response [7]. It is an agent-based model composed of three virtual spaces, the parenchymal tissue, the secondary lymph node and the lymphatic/humoral circulation. The immune response to a localized viral infection is simulated, during which an immunity gain or loss or a hyper-response might occur. The Basic Immune Simulator was updated in 2010, now including new cell types and enhanced behavioral rules [8]. Using the new version, a network analysis was applied defining the cell types as nodes and their interaction as edges.

SIMMUNE is more a modeling environment than a model of a certain phenomenon [9]. Its purpose is to investigate how context adaptive behavior might emerge from local cell-cell and cell-molecule interactions. The model is composed of a cellular automaton on a molecular level. Molecules can be defined that interact according to the behavior they get equipped with. These entities move within a discrete lattice, which can be subdivided into different compartments. Since it takes a generic approach to the modeling of cell biology, SIMMUNE is able to simulate a wide area of signal cascades, not only those related to immunology.

SIMISYS is focused on the different stages of bacterial infection [10]. The model is based on a cellular automaton and composed of two different grids representing a blood vessel and a lymph node. By means of the simulation, the authors want to learn more about the interplay between the innate and adaptive immune system.

Another rule-based study is the tumor-immune model by Mallet and De Pillis [11]. They use a hybrid form of a cellular automaton with partial differential equations. The purpose of this model is to examine the effects of cytotoxic lymphocytes and natural killer cells in the early stages of tumor growth under limited nutrient supply.

The ImmSim model has also been enlarged, refined and applied to a variety of phenomena. Castiglione et al. translated ImmSim to ANSI C language and applied their refined model, C-ImmSim, to several immunological phenomena, such as Epstein-Barr virus infection [12], TH1/2 differentiation [13], HIV-infection [14] and immune therapy of cancer [3]. C-ImmSim applied to cancer simulates a solid tumor under immune therapy, including all main mechanisms of the humoral and cellular adaptive immune response. Lollini et al. used the architecture of C-ImmSim to build SimTriplex, a model for HER2/neu transgenic mice treated with the Triplex cell vaccine [15]. This model was extended and applied to lung cancer metastasis in MetastaSim, which is a hybrid agent-based/differential equation model [16].

For our research interest of studying peptide vaccination in cancer treatment, the model C-ImmSim was the most suitable. Indeed it provides all the important behavioral rules for immune and cancer cells. The only major drawback of C-ImmSim is that the immune receptors are modeled as bit-strings, having no direct translation to amino acid sequences. Therefore, we decided to extend C-ImmSim, producing a sequence-based version, VaccImm, where all calculations depend on the amino acid sequence of immune receptors, MHCs and antigens. In VaccImm, experimental and clinical data of cancer targets, expression data or MHC-genotypes are directly integrated into the model and the simulation predicts the success of peptide vaccination taking the amino acid sequence into account.

In order to understand the architecture of the model, C-ImmSim is described in more detail in the next chapter. Afterwards, the adoption of amino acid sequences in place of bit-strings is discussed.

### C-ImmSim

Our present model VaccImm is based on C-ImmSim [3], an agent-based model written in ANSI C language. C-ImmSim is composed of a Cartesian 3D lattice that contains cancer cells and several types of immune-cells moving from one lattice point to another in discrete time steps corresponding to 8 hours of real life. The different types of immune cells can be classified as helper T-cells (TH), cytotoxic T-cells (TC), B-cells, macrophages and dendritic cells (DCs). In addition, the model includes different classes of molecules comprising antibodies, antigens, Interleukin-2 (IL-2) and a danger signal that represents a general activator signal for macrophages. These cells and molecules can interact according to behavioral rules simulating the phenomena of an adaptive immune response against cancer.

The lymph node close to the tumor is modeled explicitly by the lattice that contains all cell types and molecules. In contrast, the thymus selection is simulated implicitly, as all T-cells are probed for their reactivity against MHC and self-peptides in positive and negative selection before being introduced into the simulation.

All specific interactions of the adaptive immune response depend on the complementarity of bit-strings in C-ImmSim. To be more explicit, each immune cell receptor, MHC, antigen or MHC-presented peptide is represented by a string composed of zeros and ones, and whenever an immune receptor meets a possible ligand, the complementarity of the bit-strings defines their interaction probability. Whereas this way of representing the molecular specificity is very generic and computationally easy, it is very distant from real protein-protein interactions. The development of a sequence-based definition of molecules is therefore an important step toward the generation of more realistic models.

### From Bit-Strings to Amino Acids

Rapin et al. have extended C-ImmSim to a sequence-based version for simulating generic infectious diseases [4]. In contrast, our extension, VaccImm, is focused on peptide vaccination in cancer therapy and therefore has to fulfill several different needs.

To turn the bit-string model into a sequence-based model, amino acid sequences need to be introduced for all specific interactions between an immune receptor and a ligand. In addition, in C-ImmSim the interaction strength is based on the complementarity of bit-strings. In VaccImm, the interaction probabilities dependent on amino acid sequence had to be developed. Rapin et al. used the Miyazawa-Jernigan potential [17] for that purpose. In the present work we demonstrate that this approach does not clearly differentiate observed immune receptor-ligand complexes from random complexes. For that reason, we developed new interaction potentials specific to B-cell and T-cell receptors that are able to clearly differentiate experimentally observed from random complexes.

## Results

### Development of an Interaction Potential for Immune Receptor-Ligand Binding

An essential step in the adaptive immune response is the recognition of a target cell by the immune cell mediated by the immune receptor binding to its matching ligand. Both the interaction of a T-cell receptor with a peptide-MHC complex and a B-cell receptor with an antigen are very complex in nature and still a matter of research [18], [19]. Therefore, it is desirable to have a universal function differentiating binding from non-binding immune complexes.

**Figure 1:**
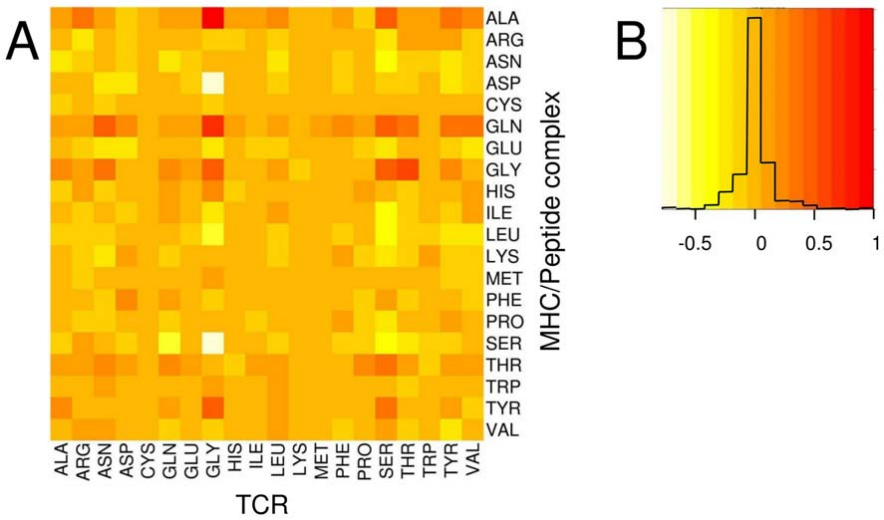
Interaction Potential IP_T_ for MHC-Peptide/TCR Complexes. A: The newly developed interaction potential IP_T_ for MHC-peptide/TCR complexes. B: Color code and color frequency for interaction potential map.

In the present study, we develop a separate empirical pair-wise interaction potential (IP) between the immune receptor and its ligand for both the B-cell and the T-cell receptors. The IP is based on an analysis of crystal structures from the Protein Data Bank [20] using a similar method to that used by von Eichborn et al. [21]. We compared the amino acid pairs within the interface of observed crystal structures of immune receptor complexes to those of random structures built to represent non-binding receptor ligand pairs. From this calculation, we gained an IP specific for the type of immune receptor-ligand-complex; IP_T_ for T-cell receptors and IP_B_ for B-cell receptors (Fig. 1 and Fig. S1).

**Figure 2:**
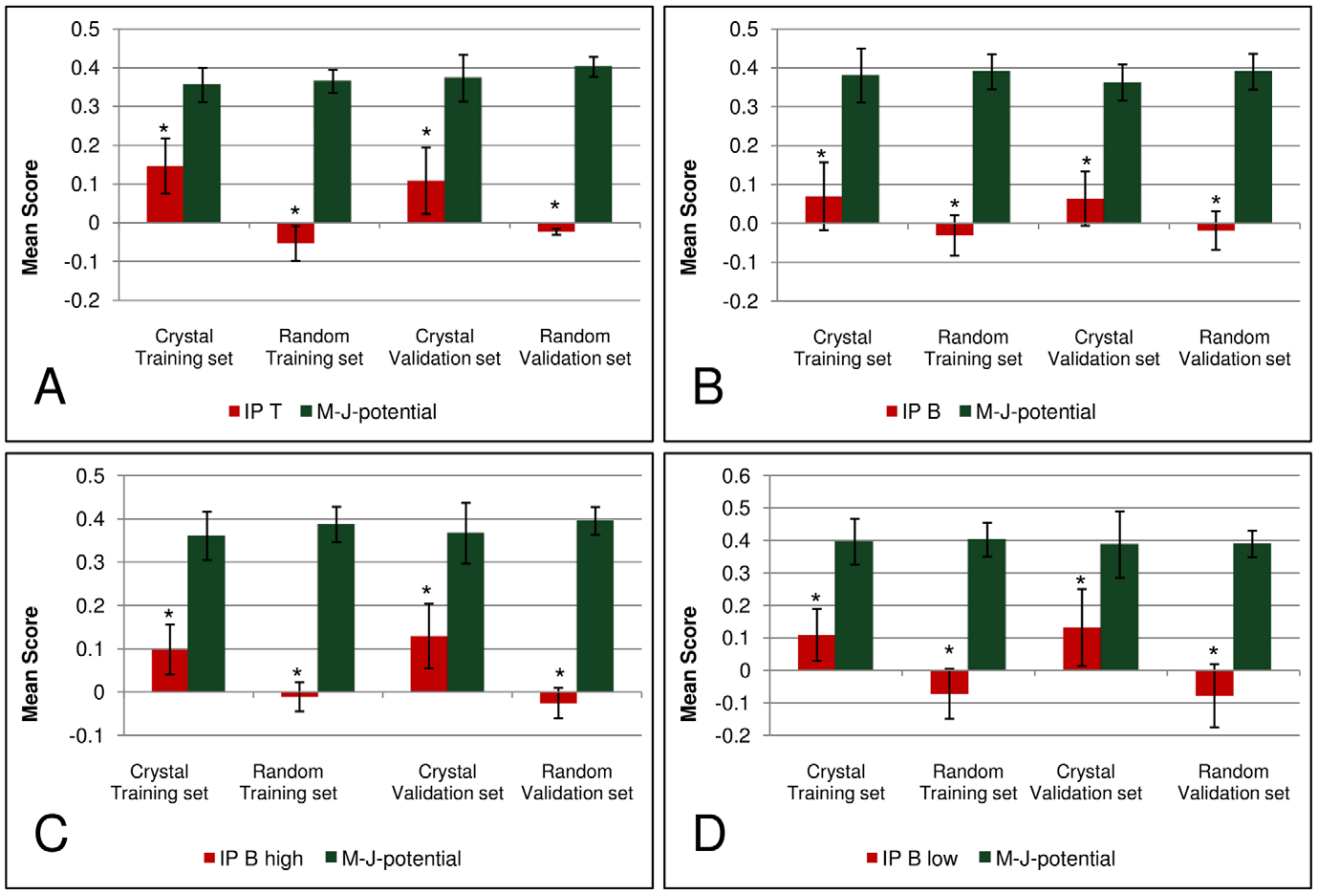
Evaluation of the Miyazawa Jernigan Potential and the Newly Developed Potentials IP_T_, IP_B_, IP_B high_ and IP_B low_. Mean scores of MHC/peptide-TCR crystal and random structures for training (90% of structures) and validation set (10% of structures) are depicted. Scores observed using the newly developed interaction potentials and the Miyazawa-Jernigan interaction potential [17] are compared. A: Scores of MHC/peptide-TCR complexes using IP_T_. B: Scores of antibody/antigen complexes (all) using IP_B_. C: Scores of antibody/antigen complexes with antigens of high glycine frequency (>6.9%) within the interface using IP_B high_. D: antibody/antigen complexes with antigens of low glycine frequency (<6.9%) within the interface using IP_B low_. * indicates P-value of crystal and random set below 0.01 using Mann-Whitney test. M-J-potential: Miyazawa-Jernigan potential.

Before the analysis, structures were separated into training (90%) and validation (10%) sets and then all complexes were scored using IP_T_ or IP_B_. The scores of training and validation sets of the crystal and artificial structures were compared to scores that were gained using the well known Miyazawa-Jernigan potential [17], as Rapin et al. [4] used the Miyazawa-Jernigan potential to score the immune receptor-ligand interaction. Our analysis shows that for the peptide/MHC-TCR interaction the Miyazawa-Jernigan potential is not able to differentiate between crystal and artificial structures, while the sets can be clearly differentiated using IP_T_ (Fig. 2A). The area under curve (AUC) was calculated with the Mann-Whitney test for the structures scored with IP_T_ yielding an AUC of 0.93 and a P-value below 0.01, so the differentiation is highly significant.

For the BCR-antigen interaction, the Miyazawa-Jernigan potential again does not differentiate between the crystal and the artificial structures while IP_B_ does (Fig. 2B). As the standard deviation is very large when using IP_B_, we looked for any characteristics of the structures to explain this phenomenon. We found that the standard deviation can be considerably decreased if the data sets are subdivided into sets with high and low glycine frequencies within the antigen interface (Fig. 2C+D and Fig. S2,S3). The limit was set at 6.9% glycine within the interface of the antigen, as this is the frequency of glycine on protein surfaces. The separation resulted in two data sets of nearly equal size. The Mann-Whitney test yields an AUC of 0.93 for the high glycine data set (IP_B high_) and 0.88 for the low glycine data set (IP_B low_), while the P-value is below 0.01 for both. The reason for this difference in high and low glycine content in the interface of an antigen might be that the higher glycine content allows sharper turns in the backbone and that could lead to a slightly different mechanism of recognition by antibodies.

### Application of the Interaction Potential in VaccImm

The newly developed IP was applied to the agent-based simulation VaccImm, an extension of C-ImmSim [3], along with approved prediction methods for MHC-peptide binding (see Materials and Methods).

**Figure 3:**
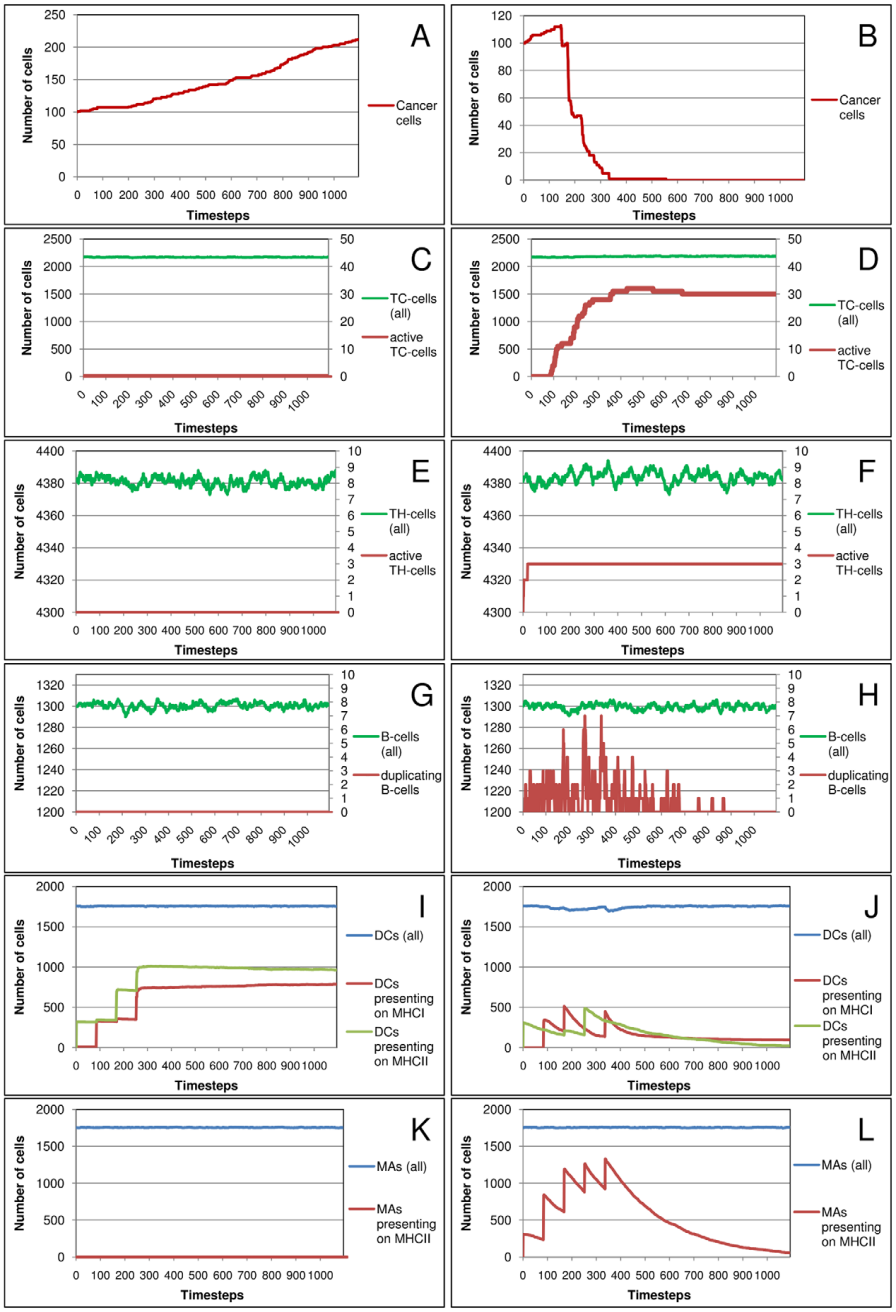
Peptide Vaccination With and Without Adjuvant. Cell counts are depicted for cancer cells (A/B), cytotoxic T-cells (C/D), helper T-cells (E/F), B-cells (G/H), dendritic cells (I/J) and macrophages (K/L) over one year of simulated time. One time step equals 8 hours. Left panel: No adjuvant was added; right panel: Adjuvant was included. For both experiments one hundred initial cancer cells were simulated within 5 µl of blood that were treated with peptide injections starting at time point zero and being repeated five times at an interval of 28 days. The MHC-genotype of this virtual individual is HLA_A80/HLA_B56/HLA_DQA2_DQB2/HLA_DRB3. The antigen A2SUH6_HUMAN (Survivin variant 3 alpha) was chosen from over-expression data in prostate tumors. Two MHC I-binding peptides and two MHC II-binding peptides were injected.

VaccImm accounts for the most important immune cell populations in their different activation states. In this section, we analyze these output-curves of the cell counts over time. Figure 3 depicts example curves of cell counts resulting from a successful (right panel) and a failing (left panel) peptide vaccination therapy. The difference between the two simulations is that the peptides of the successful treatment were emulsified in adjuvant while the peptides of the failed treatment were not. To initiate or boost the immune response, adjuvant is often added for immunization [22]. Cancer epitopes are naturally of low immunogenicity as they result from the body's own proteins that are usually not recognized as foreign.

Without adjuvant, the cancer cells double within one year (Fig. 3A), while a successful treatment is able to eradicate the tumor within less than four months (Fig. 3B). In successful immune therapy, matching T-cell and B-cell receptors recognize the antigen and TC-cells, TH-cells and B-cells get activated and replicate, as seen in Figure 3. In contrast, peptide vaccination without adjuvant is not able to activate TH-cells, TC-cells nor B-cells for proliferation. Also the antigen presenting cells (APCs) behave differently in the two settings. In both cases, DCs present peptides on their MHC I and MHC II, but without a stimulating signal from the adjuvant this leads to anergy rather than to activation of the T-cells. Macrophages are not recruited to present peptides on their MHC II without adjuvant stimulation. In successful treatment, presentation of both APC types on MHC peaks when the antigen is injected but cell counts decrease immediately afterwards because activated TC-cells kill the APC.

### Impact of IP_T_ on the Simulation with VaccImm

The most important step of the immune response against a tumor is the recognition of a cancer epitope on MHC I by TC-cells that will result in the lysis of tumor cells under activating conditions. However, the bottleneck of cancer immunotherapy is that most of the TC-cells that could possibly recognize the cancer cells are eliminated in the thymus as they are reactive against self-peptides. If a matching TC-cell survives thymus selection, lowering of the tumor burden by the TC-cell clone depends on several other factors e.g. the MHC-binding properties of the cancer epitope, the way of antigen encounter by the TC-cell, its spatial environment and the cytokine interplay. To analyze the impact of IP_T_ and thymus selection on the simulation, we compared their calculated reactiveness.

**Figure 4:**
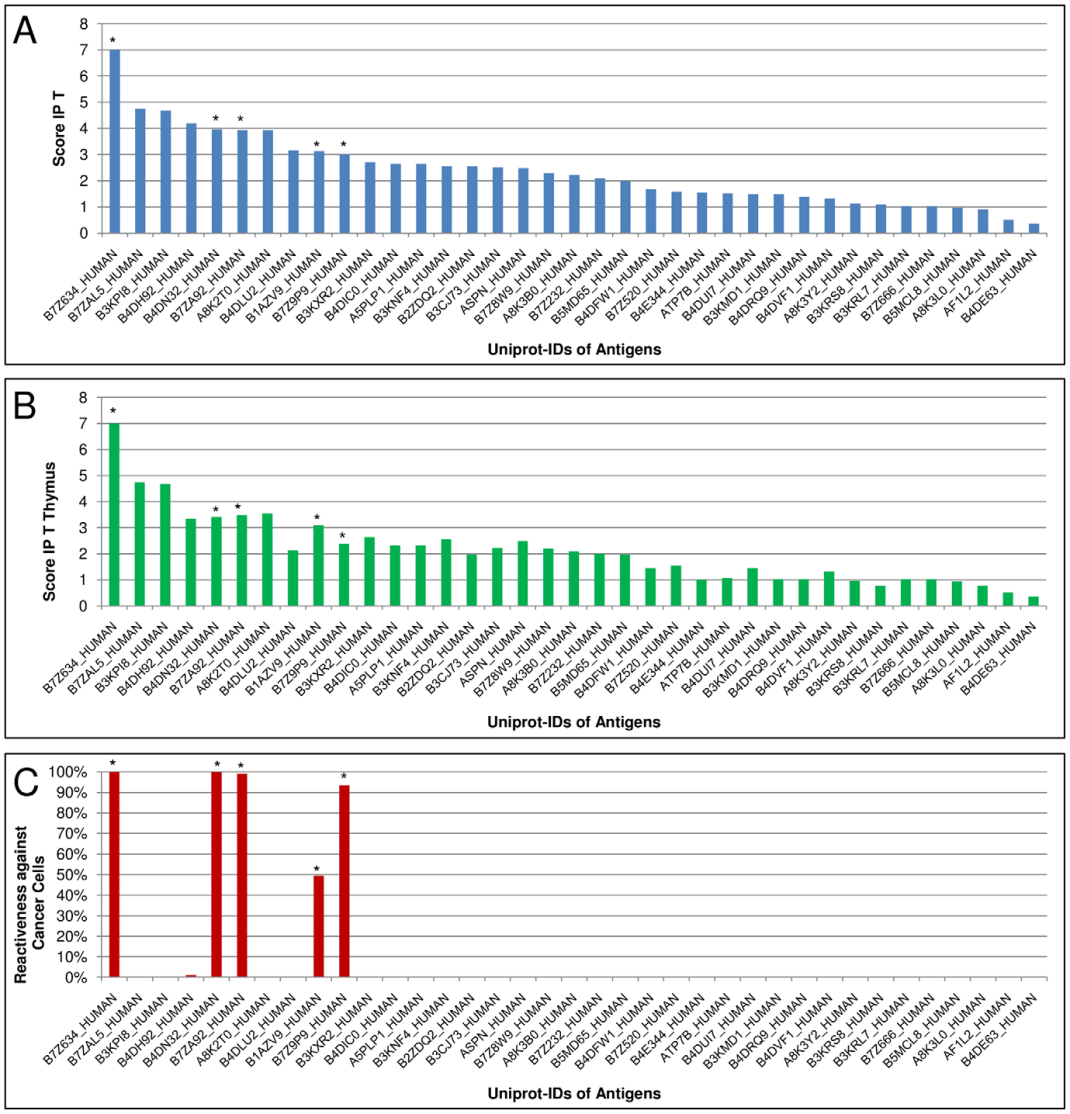
Comparison of IP_T_, Thymus Selection and Simulation. Scores for different antigens using IP_T_ are compared with (A) a random sample of about 15,000 TCRs , (B) all TCRs from the set that survived the thymus selection and (C) with the reactiveness against cancer cells in the simulation with VaccImm. The antigens in all three diagrams are sorted by their IP_T_-score including all TCRs. Antigens inducing an immune response against the tumor, are marked with *. Simulation data were obtained from 100 simulations per column. The MHC-genotype is HLA_A02/HLA_DRB3. For all experiments one hundred initial cancer cells were simulated within 5 µl of blood that were treated with peptide injections starting at time point zero and being repeated five times at an interval of 28 days. The antigens were chosen from over-expression data in prostate tumors. One MHC I-binding peptide and one MHC II-binding peptide were injected.

Different antigens were evaluated for their score using IP_T_ and the T-cell reactivity they induced in the agent-based model simulation. For that purpose, a set of about 15,000 TCR sequences was randomly generated. In the first step, the antigens were sorted by the highest IP_T_- score they gained when probed against the whole TCR set (Fig. 4A). In the second step, all TCRs that would not survive thymus selection, because of their complementarity to self peptides, were eliminated and the antigens were scored again with the reduced TCR set (Fig. 4B). In the third step, the entire simulation was executed for all the antigens using VaccImm (Fig. 4C). The reactiveness against cancer cells was defined as the reduction of tumor size compared to unlimited growth, with 100% reactiveness leading to complete tumor eradication.

In thymus selection, strongly self-reactive TC-cells are eliminated, that is why several scores are lowered after thymus selection (Fig. 4A+B). Nevertheless, the same overall trend was observed for stronger and weaker immunogenic antigens. In the simulation, only very few antigens are able to induce a sufficient immune response to reduce or eradicate the tumor (Fig. 4C). Antigens having a low score from IP_T_ are not recognized by TC-cells because of their low interaction probability. However, some antigens with high interaction probabilities calculated from IP_T_ are also not able to induce a sufficient immune response. This result underlines that the complete immune cell and signal molecule interplay needs to be investigated for understanding the tumor-immune-interaction.

In the next section, we analyze the failing antigen treatments in more detail.

### Reasons for Failures in Treatment

**Figure 5:**
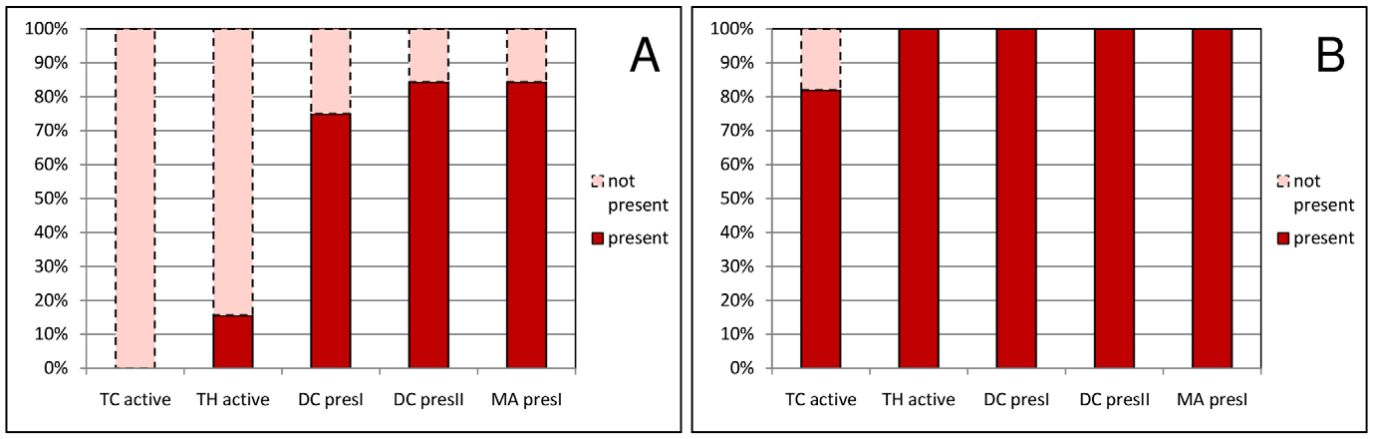
Reasons for Failures in Treatment. The propensity to obtain certain activation states of the immune cell populations is depicted. Experiments are grouped into frequent failures (A) that induce a sufficient immune response in less than 50% of the simulations and rare failures (B) with more than 50% sufficient responses. Abbreviations: TC active: active cytotoxic T-cells. TH active: active helper T-cells. DC presI/DC presII: dendritic cells presenting peptides on MHC I/MHC II. MA presII: macrophages presenting peptides on MHC II.

In order to better understand the reasons why the immune system failed to successfully fight the tumor for most of the antigen injections, we took a closer look at the immune cell populations (Fig. 5). A successful treatment was defined as a simulation with a smaller tumor size after one year of simulated time with respect to the beginning of treatment.

Interestingly, we found that the resulting patterns can be separated into two groups: In the first group, a sufficient immune response was induced in less than 50% of the simulations (frequent failures, Fig. 5a) and in the second group in more than 50% (rare failures, Fig. 5b). In both groups, peptide presentation on MHC I and II by DCs and macrophages was successful in 75% to 100% of the simulations. Consequently, this part of the immune response was not the main bottleneck in the treatment. In the frequent failures, no active TC-cells and almost no active TH-cells were observed. For the rare failures, active TC-cells were observed in more than 80% of the failing simulations. We did take a closer look at these TC-cell populations, searching for a reason why they were not able to eradicate the tumor. We found that these TC-cell populations were either very small (composed of only one or two cells) or started dividing at a later stage of the simulation such that the tumor size could not be decreased before the end of the simulation (data not shown).

These observations indicate that the rare failures would occur with an even lower frequency if the simulations were carried out over a longer time period. In contrast, the frequent failures are prone to failing to induce an immune response either because their antigens are too closely related to self-peptides or because the TC-cells did not encounter the antigen in a sufficiently activating environment.

### Simulation Run Time and Error Propagation from the Interaction Potential

VaccImm is able to simulate up to one million initial cancer cells treated with a maximum of ten peptides. One run of the simulation takes from several seconds to a few minutes on a single core of a 3 GHz Intel Core 2 Duo CPU depending on the input parameters chosen.

Before starting the simulation, peptide sequences binding to MHC I and MHC II are predicted within a few seconds. The VaccImm simulation itself can be divided into two parts, thymus selection of initial population of T lymphocytes and spatial dynamics within the agent-based model accounting for the whole immune response. Having a simulation with standard configuration (see Table 1), the two parts require about the same amount of CPU time. In general, however, while the time for thymus selection increases with the number of self-peptides and the number of different MHC-alleles, the time for the agent-based simulation increases with cell numbers, simulated time and volume. Some benchmark experiments with their simulation times are given in Table 1.

**Table 1:** Computational Cost of the Simulation.

Configuration	Computational Time [s]
Standard*	21
Initial tumor size [cells]: 10 000	33
Initial tumor size [cells]: 100 000	129
Initial tumor size [cells]: 1 000 000	855
Simulated Space [µl]: 10	82
Simulated Space [µl]: 20	165
Simulated Space [µl]: 20, Initial tumor size [cells]: 100 000	250
2 MHC-I binding peptides, 2 MHC-II binding peptides	40
3 MHC-I binding peptides, 3 MHC-II binding peptides	73
4 MHC-I binding peptides, 4 MHC-II binding peptides	100
5 MHC-I binding peptides, 5 MHC-II binding peptides	117
2 MHC-I alleles, 2 MHC-II alleles	31
3 MHC-I alleles, 3 MHC-II alleles	57
6 MHC-I alleles, 6 MHC-II alleles	45
2190 time steps	25
4380 time steps	34

*The standard configuration for the simulations is: Uniprot-ID 2B1D_HUMAN, 1 MHC-I allele, 1 MHC-II allele, 1 MHC-I binding peptide, 1 MHC-II binding peptide, 5 µl simulated space, 1095 time steps, 100 cancer cells as initial tumor size. All changes to this configuration are mentioned in the table.

**Figure 6:**
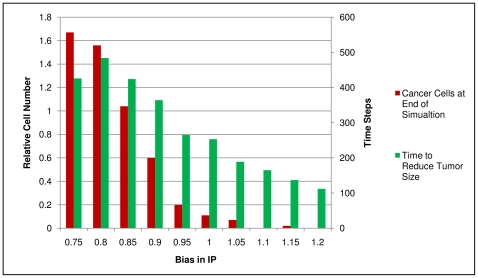
Error Propagation from IP to Simulation. The relative number of cancer cells after one year of treatment compared to the tumor size at the beginning of the simulation is depicted (red bars) along with the average number of time steps needed to reduce the tumor size (green bars). All interaction probabilities in the simulation calculated with IP were multiplied with the bias in IP shown on the y-axis. Data were obtained from 100 simulations per column. The MHC-genotype is HLA_A02/HLA_DRB1-1. For all experiments one hundred initial cancer cells were simulated within 5 µl of blood that were treated with peptide injections starting at time point zero and being repeated five times at an interval of 28 days. The antigen B7Z8B0_HUMAN was chosen from over-expression data in prostate tumors. One MHC I-binding peptide and one MHC II-binding peptide were injected.

To investigate the error propagation from the IP to the simulation, an example was chosen where the tumor is almost eradicated. The interaction probability calculated using IP_T_, IP_B high_ and IP_B low_ was increased or decreased in a stepwise manner and the impact on the final number of cancer cells and the time it takes to reduce the cancer cell number compared to the initial tumor size was analyzed (Fig. 6). Increasing the interaction probability led to a gradual decrease in the time to reduce the tumor size while the tumor was almost or completely eradicated with an increase in the interaction probability of 10% and more. Decreasing the interaction probability increased the time to reduce the tumor and the relative number of cancer cells at the end of the simulation. At a 15% decrease of the interaction probability, the mean number of cancer cells was not reduced compared to the beginning of the simulation. Decreasing the interaction probability by 20% or more leads to tumor reduction in less than 50% of the simulations.

From these results we can deduce that a bias in IP_T_, IP_B high_ and IP_B low_ will affect the results of the simulation. A qualitative difference in the outcome, as having or not having an induction of the immune response, will probably occur from a bias larger than 10–15%.

## Discussion

In this work, we developed new IPs between an immune receptor and its ligand from a statistical analysis of crystal structures for B-cell and T-cell receptors. It was shown that the newly developed IPs are able to differentiate naturally observed from randomly generated immune complexes, in contrast to the Miyazawa-Jernigan potential [17]. We believe that our empirically developed potentials account for specific properties of the respective immune receptor interactions, that are not included in general potentials like the Miyazawa-Jernigan potential.

In the second step, the newly developed IPs were applied to the agent-based model VaccImm, an extension of C-ImmSim [3]. There is one other agent-based model that includes the amino acid sequence in calculation; the model of Rapin et al. [4] simulates the immune response against infections and was developed independently from VaccImm. While Rapin et al. took a more general approach to infectious diseases; we focused on the specific needs of peptide vaccination in cancer therapy.

Prediction methods for complex protein-protein interactions, such as our newly developed IPs, always have a limited accuracy. Therefore, we investigated what impact a systematic error in the IPs would have on the VaccImm simulation. As demonstrated, a bias in the IPs will surely affect the simulation, but qualitative changes in the outcome are not expected as long as the bias in the interaction prediction stays below 10%.

In contrast to the former versions of C-ImmSim, and similarly to the new published version [4], VaccImm predicts reactivity of the immune system against real amino acid sequences of any injected peptide. The model includes several core phenomena of the immune system, e.g., the humoral and cellular branch, clonal expansion of single immune cells, thymus selection of T-cells and spatial interactions of autonomous cells with distinct specificity. Still, some properties of the immune-cancer interplay might be underrepresented in the current version of the model and we are planning to extend VaccImm in that direction. The most important features we are planning to include in the future are the different types of cytokines, the influence of regulatory T-cells (T_reg_s) and the mutation of cancer cell epitopes.

Cytokines have an essential role in facilitating communication between immune cells, most of them acting within short ranges [23]. Thus far, only IL-2 and a general danger signal are included in the simulation, but agent-based models are particularly tailored to take into account local effects of molecules in a spatial environment. Consequently, we are planning to include a more detailed representation of cytokines in the model in the future. Examples for important cytokines in the cancer-immune interplay to be included soon are the transforming growth factor-β [24] or interferon-γ [25].

T_reg_s are an immune cell population suppressing the immune response, presumably with the main function to prevent autoimmune diseases [26]. Their immunosuppressive effect hinders the immune response against cancer [27]. Therefore, our plan is to include this cell type in the next version of VaccImm.

Our current model does not account for mutation or changes in gene expression of the tumor. The genome of a tumor is often unstable and many different mutations or gene rearrangements result in a huge diversity of tumor cells that do not present the same epitopes on their MHCs. It is frequently observed, particularly under treatment, that some tumor cells change their behavior or their expression pattern, thereby circumventing eradication; a phenomenon called tumor escape [28]. For this purpose, we plan to include tumor epitope mutation and changes in the antigen expression levels.

The present model is a first step towards *in silico* experiments predicting T-cell reactivity taking into account the amino acid sequence. Expanding knowledge in tumor immunology will help to further improve our model. As VaccImm is very flexible and its architecture is separated into different modules, it can be easily extended or refined with new experimental data.

## Materials and Methods

### Peptide Binding to MHC Complexes

The first step necessary for T-cell recognition is the processing of a protein to a peptide and its presentation on a MHC complex by the target cell. This process has been evaluated in great detail by several research groups. It has been found that key residues exist at distinct places within the peptide that are most important for binding to the MHC [29]. From the accumulated MHC binding and elution data, several methods have been generated to predict which part of the protein sequence will be presented by a certain MHC. For simulation, we used two well-known position-specific scoring matrices to predict peptide-MHC binding, namely *smmpmbec* (http://tools.immuneepitope.org) for MHC I binding and *arb* [30] for MHC II binding.

### Development of an Interaction Potential for Immune Receptor-Ligand Binding

A separate empirical pairwise interaction potential between the immune receptor and its ligand was developed for both the B- and T-cell receptor, based on crystal structures from the Protein Data Bank [20] using a similar method to that used by von Eichborn et al. [21]. A statistical analysis was drawn from 237 antibody-antigen and 33 MHC-TCR non-redundant crystal structures. Interacting amino acid pairs between receptor and ligand were defined as all residues having no more than 8 Å distance between their Cα atoms. We compared the observed crystal structures to random structures built to represent non-binding receptor ligand pairs. To generate the random structures, the peptide sequences of the MHC-peptide complex or the antigen were replaced by random sequences with the same amino acid distribution that was observed on general protein surfaces. The number of interacting pairs was counted for both the crystal and the random structures and the numbers were subtracted using the formula:
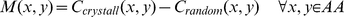
where M is the interaction potential scoring matrix and C_crystal_ and C_random_ are the counts for crystal and random structures, respectively. From this calculation, we gained an interaction potential (IP) specific for the type of immune receptor-ligand-complex; IP_T_ for T-cell receptors and IP_B_ for B-cell receptors. Within IP_T_ and IP_B_, positive values represent an increased likelihood of observing the corresponding amino acid pair in the interface of the complex, while negative values represent a decreased likelihood. IP_T_ and IP_B_ were normalized to a maximum value of 1.0 and then used to score the crystal and artificial structures for validation.

### Application of the Interaction Potential to the Simulation

Whenever an active T-cell meets an APC presenting a peptide on a matching MHC type, their interaction probability is calculated using IP_T_. To define which residues of the immune receptor and the ligand are in contact with each other, we created contact matrices from crystal structures analogously to the work of Rapin [4]. Again, the interacting amino acid pairs between receptor and ligand were defined as all residues having at most 8 Å distance between their Cα atoms. For each crystal structure, a matrix composed of ones and zeros representing interacting and non-interacting residues was created. In a next step the interaction probability was calculated as the sum of the IP_T_ matrix values for all interacting residues that is normalized by the number of interactions.

As the number of MHCs with known structure is limited, MHC sequences not having been crystallized were mapped to the closest related MHC structures for the definition of the contact residues. Contact matrices were built from MHC-peptide-TCR complexes containing HLA_A02 (pdb-ID: 1OGA), HLA_B08 (1MI5), HLA_B35 (3MV7), HLA_B44 (3DXA), and HLA_DRB1-4 (1J8H). For the interaction between a BCR or an antibody and its antigen, we used one high-resolution structure of an antibody binding to a peptide (2B1H) to create the contact matrix, as the injected antigens are peptides and their tertiary structure can probably be neglected.

### Steps of the Simulation

Starting the simulation, the different behaviors and movements of the cells are executed in discrete time steps. For the purpose of modeling peptide vaccination to treat cancer with VaccImm, the timeline of observed phenomena is as follows:

1. The peptides are injected emulsified in adjuvant. The adjuvant is modeled as a danger signal activating macrophages.
2. APCs take up the peptides by phagocytosis. In the case of macrophages and DCs, phagocytosis is unspecific, while B-cells have to recognize the antigen for ingestion. The probability of recognition is calculated using the B-cell contact matrix and IP_B high_ or IP_B low_ depending on the antigen.
3. Peptides are processed and presented by the APCs. The probability of presenting a certain peptide by a specific MHC is calculated using the position-specific scoring matrices described above.
4. The peptides presented on MHC I and MHC II can be recognized by TC- and TH-cells, respectively. The probability for recognition is calculated using IP_T_ together with the contact matrix for the respective MHC. Reacting T-cells will be activated to duplicate and create memory cells.
5. The humoral and the cytotoxic immune response begin. Activated TC-cells might recognize the cancer cells, while the probability of interaction is calculated again via IP_T_ and the respective contact matrix. Successful interactions between TC-cells and cancer cells will kill the cancer cells and therefore the tumor will be eliminated. B-cells stimulated by TH-cells start duplicating into memory cells and antibody-producing plasma cells. Plasma cells secrete antibodies that clear the antigen.

This model is quite generic and flexible and captures the main mechanisms in the tumor-immune interaction. As an output of the model, cell lines and their respective activation states can be followed over time.

### Input Data

For VaccImm, all sequence-based input parameters needed to be changed with respect to C-ImmSim. Within the present analysis, we focus on urological tumors of kidney and prostate, but the program is very general and able to simulate any solid tumor. As input, the model needs the amino acid sequences of the cancerous proteins, the MHC alleles, and the self-peptides used in thymus selection.

Cancerous proteins were selected based on expression data from patients suffering from prostate or kidney tumors. [31]. Any gene that is over-expressed at least 2 times in the analyzed cancerous tissue with respect to healthy control tissue was chosen as a cancer target. The respective protein sequences were collected from the UniProt. [32]. Peptides presented on MHC I or MHC II originating from the protein sequences were predicted using prediction algorithms (*consensus* [33] for MHC I, *smm_align* [34] for MHC II) from the Immune Epitope Database [35]. These predicted peptides are presented by the MHCs in the simulation. The same peptide sequences are used for injection in immune therapy within the simulation. As short peptides usually do not fold into complex secondary structures, the injected peptides are used directly as epitopes for recognition by B-cells and antibodies.

One part of the simulation is the selection of T-cells within the thymus. If any T-cell receptor is strongly reactive to self-peptides, the T-cell will be eliminated. To define the self-peptides, we downloaded all peptide sequences eluted or known to bind to MHC I or MHC II from the IEDB [35]. To decrease the number of self-peptides and therefore increase the speed of the simulation, we used only the 50 self-peptide sequences most similar to each injected peptide. All other self-peptide sequences having a lower similarity will not interfere with the injected peptides and thus are neglected.

The MHC sequences for all different alleles were downloaded from the UniProt [32], 61 for MHC I and 20 for MHC II. All TCR and BCR sequences were generated at random when the respective cell was introduced into the simulation.

Any other parameter not pertaining to the amino acid sequence implementation are left unchanged with respect to C-ImmSim [3].

## Supporting Information

Figure S1 **Interaction Potential IP_B_ for All Antibody/Antigen Complexes.** A: The newly developed interaction potential IP_B_ for all antibody/antigen complexes. B: Color code and color frequency for interaction potential map.
(TIFF)

Figure S2 **Interaction Potential IP_B high_ for Antibody/Antigen Complexes, High Glycine Frequency.** A: The newly developed interaction potential IP_B high_ for antibody/antigen complexes with antigens of high glycine frequency (>6.9%) within the interface. B: Color code and color frequency for interaction potential map.
(TIFF)

Figure S3 **Interaction Potential IP_B low_ for Antibody/Antigen Complexes, Low Glycine Frequency.** A: The newly developed interaction potential IP_B low_ for antibody/antigen complexes with antigens of low glycine frequency (<6.9%) within the interface. B: Color code and color frequency for interaction potential map.
(TIFF)
