# Supplementary figures and images for: Development of Immune-Specific Interaction Potentials and Their Application in the Multi-Agent-System VaccImm
Source: PLoS One. 2011 Aug 17;6(8):e23257. doi: 10.1371/journal.pone.0023257 (PMC3157361; doi:10.1371/journal.pone.0023257)

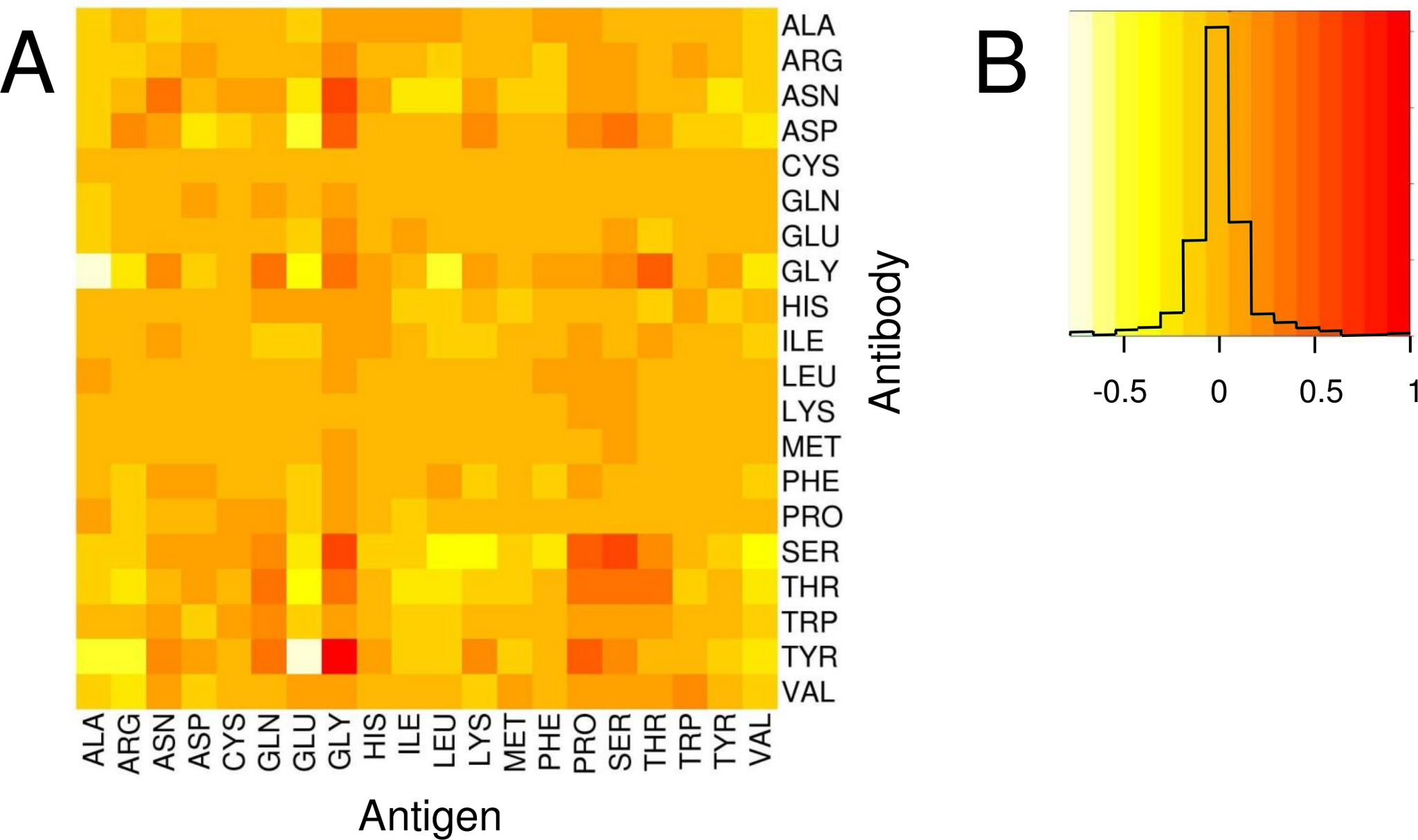

Supplement: Figure S1 — Interaction Potential IPB for All Antibody/Antigen Complexes. A: The newly developed interaction potential IPB for all antibody/antigen complexes. B: Color code and color frequency for interaction potential map. (TIFF) [file pone.0023257.s001.tif]

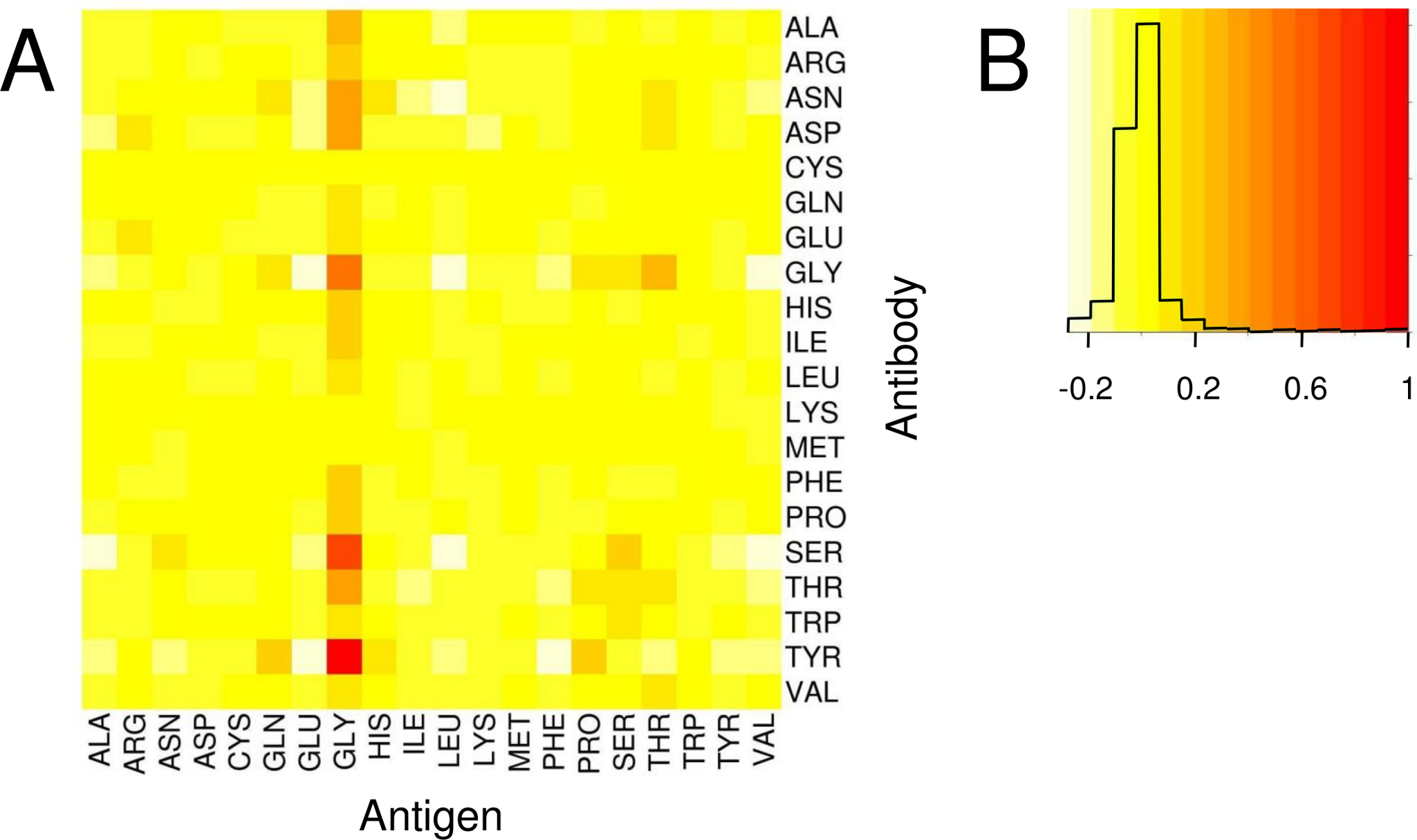

Supplement: Figure S2 — Interaction Potential IPB high for Antibody/Antigen Complexes, High Glycine Frequency. A: The newly developed interaction potential IPB high for antibody/antigen complexes with antigens of high glycine frequency (>6.9%) within the interface. B: Color code and color frequency for interaction potential map. (TIFF) [file pone.0023257.s002.tif]

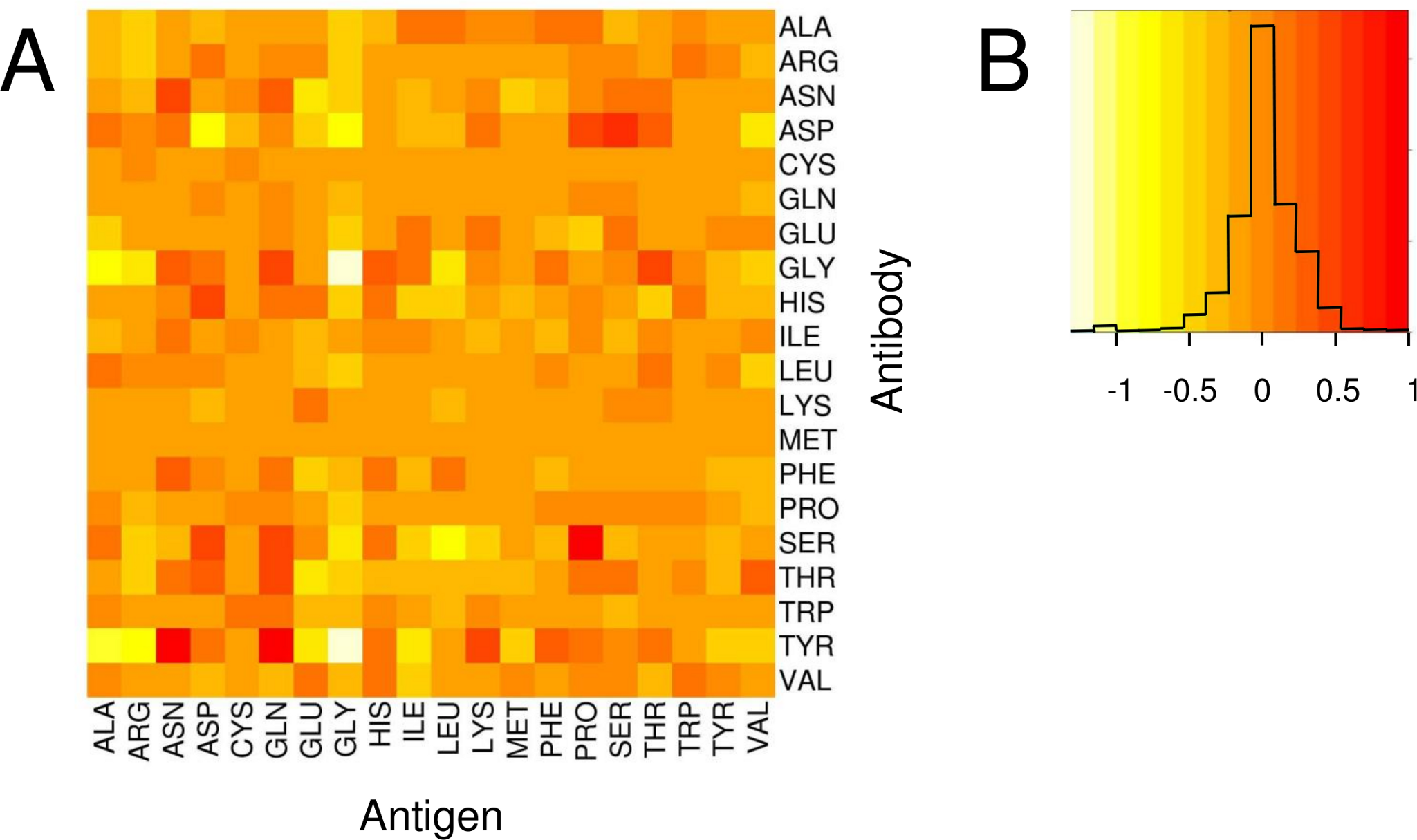

Supplement: Figure S3 — Interaction Potential IPB low for Antibody/Antigen Complexes, Low Glycine Frequency. A: The newly developed interaction potential IPB low for antibody/antigen complexes with antigens of low glycine frequency (<6.9%) within the interface. B: Color code and color frequency for interaction potential map. (TIFF) [file pone.0023257.s003.tif]
